# Beyond Angiogenesis: The Multitasking Approach of the First PEGylated Vascular Endothelial Growth Factor (*Cdt*VEGF) from Brazilian Rattlesnake Venom

**DOI:** 10.3390/toxins15080483

**Published:** 2023-07-31

**Authors:** Isabela Ferreira, Isadora Oliveira, Karla Bordon, Mouzarllem Reis, Gisele Wiezel, Caroline Sanchez, Luísa Santos, Norival Santos-Filho, Manuela Pucca, Lusânia Antunes, Daiana Lopes, Eliane Arantes

**Affiliations:** 1 Department of BioMolecular Sciences, School of Pharmaceutical Sciences of Ribeirão Preto, University of Sao Paulo, Ribeirao Preto 14040-903, SP, Brazil; 2 Department of Clinical Analysis, Toxicology and Food Science, School of Pharmaceutical Sciences of Ribeirao Preto, University of Sao Paulo, Ribeirao Preto 14040-903, SP, Brazil; 3 Institute Multidisciplinary in Health, Federal University of Bahia, Vitoria da Conquista 40110-909, BA, Brazil; 4 Department of Biochemistry and Organic Chemistry, Chemistry Institute, Sao Paulo State University (UNESP), Araraquara 14800-901, SP, Brazil; 5 Department of Clinical Analysis, Sao Paulo State University (UNESP) Araraquara 14800-901, SP, Brazil

**Keywords:** vascular permeability, VEGF, *Crotalus durissus terrificus*, PEGylation, neutrophilic recruitment

## Abstract

A pioneering study regarding the isolation, biochemical evaluation, functional assays and first PEGylation report of a novel vascular endothelial growth factor from *Crotalus durissus terrificus* venom (*Cdt*VEGF and PEG-*Cdt*VEGF). *Cdt*VEGF was isolated from crude venom using two different chromatographic steps, representing 2% of soluble venom proteins. Its primary sequence was determined using mass spectrometry analysis, and the molecule demonstrated no affinity to heparin. The Brazilian crotalid antivenom recognized *Cdt*VEGF. Both native and PEGylated *Cdt*VEGF were able to induce new vessel formation and migration, and to increase the metabolic activity of human umbilical endothelial vascular cells (HUVEC), resulting in better wound closure (~50% within 12 h) using the native form. *Cdt*VEGF induced leukocyte recruitment to the peritoneal cavity in mice, with a predominance of neutrophil influx followed by lymphocytes, demonstrating the ability to activate the immune system. The molecule also induced a dose-dependent increase in vascular permeability, and PEG-*Cdt*VEGF showed less in vivo inflammatory activity than *Cdt*VEGF. By unraveling the intricate properties of minor components of snake venom like svVEGF, this study illuminates the indispensable significance of exploring these molecular tools to unveil physiological and pathological processes, elucidates the mechanisms of snakebite envenomings, and could possibly be used to design a therapeutic drug.

## 1. Introduction

The utilization of toxins as therapeutic and biotechnological assets has been steadily rising in popularity. Nevertheless, it is known that one of the most difficult challenges to overcome is to reduce the immunogenicity of exogenous proteins. In the drug development field, PEGylation stands out as a widely recognized and firmly established strategy for enhancing the pharmacokinetic and pharmacodynamic properties of biopharmaceuticals while simultaneously mitigating their immunogenicity [1]. In this specific scenario, the advent of technologies that effectively mitigate or eradicate these challenges holds great value, as these technologies enable the widespread utilization of biopharmaceuticals in therapeutic applications [2]. Since its approval by the FDA in 1990, this strategy has gained substantial traction and is now extensively employed [3]. There are 19 PEGylated biotherapeutic agents available on the market, showcasing the widespread adoption and success of this approach [4]. Regardless of the numerous advantages of PEG bioconjugation, in the domain of animal toxins, its utilization remains largely disregarded.

Snake venoms have complex and diverse mixtures of therapeutic active proteins and polypeptides. Among those, some exhibit enzymatic features such as phospholipases A_2_ (PLA_2_), L-amino acid oxidase (LAAO) [5], metalloproteases, and serine proteases [6,7], whereas others are considered to be nonenzymatic factors. Throughout the years, several nonenzymatic proteins found in snake venoms have been extensively identified and studied, revealing their classification into distinct families of structural and functional proteins. These protein families, including C-type lectins and related proteins, cysteine-rich secretory proteins (CRISPs), anticoagulant proteins, and vascular endothelial growth factors (VEGFs), play significant roles in various physiological processes and possess considerable clinical applications [8,9,10,11,12].

The World Health Organization has officially recognized envenomings caused by snakes as a neglected tropical disease [13]. Snakes from the *Crotalus* genus are responsible for approximately 2.500 snakebite envenoming cases in Brazil [14] and are represented by the *Crotalus durissus* species. In Brazil, the *Crotalus durissus terrificus* (*Cdt*) subspecies thrives as one of the prevailing rattlesnake species, primarily inhabiting the expansive Brazilian cerrado (tropical savanna) as well as open areas that have been altered by human activities [15]. *Cdt* venom is mainly composed of crotamine and crotoxin, but it may also present other components, such as serine and metalloproteases, C-type lectins, disintegrins, hyaluronidase, LAAO, vasoactive peptides, phospholipase inhibitors (PLI inhibitors), phosphodiesterases, nerve growth factors (NGF), and VEGFs [16].

In mammals, it is known that VEGF plays a critical role in the control of vasculogenesis, angiogenesis, and vascular permeability [13]. This protein class comprises eight members: (i) humans: VEGF-A (with several isoforms), VEGF-B, VEGF-C, and VEGF-D; (ii) parapoxivirus: VEGF-E; (iii) snake venoms: VEGF-F; (iv) platelet-derived growth factor: PDGF; and (v) endocrine gland-derived vascular endothelial growth factor: EG-VEGF [17].

VEGF-Fs, also named as snake venom VEGFs (svVEGFs) [18], are distinct from mammalian VEGFs due to the variations observed at a structural level in the receptor-binding domains [19,20,21]. In response to several VEGFs, there are three VEGF tyrosine kinase receptors: the fms-like tyrosine kinase-1 (Flt-1 also known as VEGFR-1) [22], the KDR (VEGFR-2) [23], and the flt-4 (VEGFR-3) receptors [24]. While the precise mechanisms underlying their involvement in signaling remain partially understood, KDR (kinase insert domain receptor) seems to facilitate three primary functions of VEGFs, namely, vascular permeability, cell migration, and proliferation [25]. To date, svVEGFs have demonstrated exceptional selectivity in binding to KDR, showcasing an affinity comparable to that of VEGF-A_165_ [26,27].

Even though their precise contribution to envenoming remains poorly understood, intriguing biological activities have been attributed to svVEGFS, e.g., they have demonstrated in vitro induction of angiogenesis [28], cardioprotective functions [29], and vascular permeability, which authors speculate as their role during snakebite envenoming [26,30,31]. In fact, this vascular permeability is already well-established for human VEGF-A and can be correlated with the interaction of leukocytes with the vessel wall; furthermore, during acute inflammatory response, VEGF-A performs the recruitment of leukocytes, which is a critical process for immune responses [32]. The involvement of svVEGFs in pivotal biological processes strongly indicates their potential as valuable assets for the advancement of novel therapeutic approaches.

On the other hand, due to their low abundance in snake venom [16,33,34], svVEGF application as a molecular tool or therapeutic drug is a challenge. In the present study, we conducted the biochemical characterization, chemical modification (mPEG-maleimide), and functional evaluation of *Cdt*VEGF from *Cdt* snake venom previously identified by our group [16], presenting a pioneering report on leukocyte recruitment.

## 2. Results

**Figure 1:**
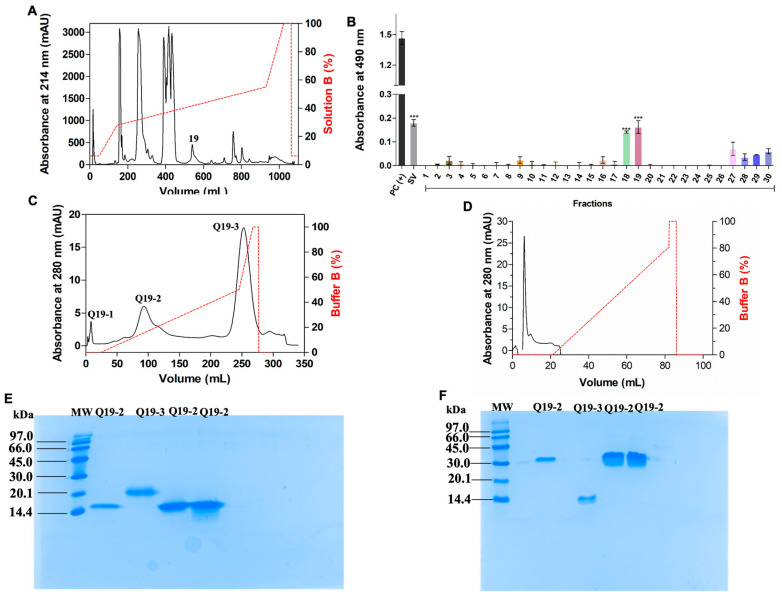
*Cdt*VEGF isolation. (**A**) Chromatographic profile of crude *C. d. terrificus* venom fractionation on C18 semipreparative column. The red dotted line represents the gradient of solution B (%). (**B**) ELISA assay using anti-VEGF-F of 30 fractions from *C.d.terrificus* fractionation. Each fraction was incubated with commercial anti-VEGF-F and absorbance was measured at 490 nm. Data are presented as mean ± SEM analyzed by one-way ANOVA and Tukey’s multiple comparison test (quadruplicate assay). *** *p* < 0.0001 when compared to its negative control. PC(+): wells coated with commercial rVEGF (AR31064PU-L, OriGene Technologies); SV: *C. d. terrificus* venom incubated with commercial anti-VEGF-F. (**C**) Fraction 19 applied on anion–ion exchange column. (**D**) *Cdt*VEGF applied on heparin affinity column. The red dotted lines represent buffer B and/or solution B (%) gradients. An amount of 15% SDS-PAGE of fractions Q19-2 and Q19-3 under reduced (**E**) and nonreduced (**F**) conditions, stained with Coomassie Brilliant Blue G-250. MW: molecular weight marker (97.0–14.4 kDa).

Native *Cdt*VEGF was isolated by two chromatographic steps. *C. d. terrificus* venom was fractionated on a semipreparative C18 column from which 30 fractions were eluted (Figure 1A). Afterwards, an indirect ELISA assay using anti-VEGF-F was performed (Figure 1B) and VEGF was detected in fractions 18 and 19. According to previous studies [16], fraction 18 is composed mainly of PLA_2_, which would make the purification of *Cdt*VEGF difficult and which also presents low recovery. Therefore, fraction 19 was chosen to be applied on an anion–ion exchange column, and three fractions were collected (Figure 1C). After, to determine the affinity to heparin of *Cdt*VEGF, it was submitted to a HiTrap Heparin column, and according to Figure 1D, *Cdt*VEGF was not capable of binding to heparin. SDS-PAGE analyses (Figure 1E,F) indicated that *Cdt*VEGF migrated as a dimer. After the purification process, *CdtV*EGF corresponded to 2% of soluble proteins from *C. d. terrificus* venom (Table 1).

**Figure 2:**
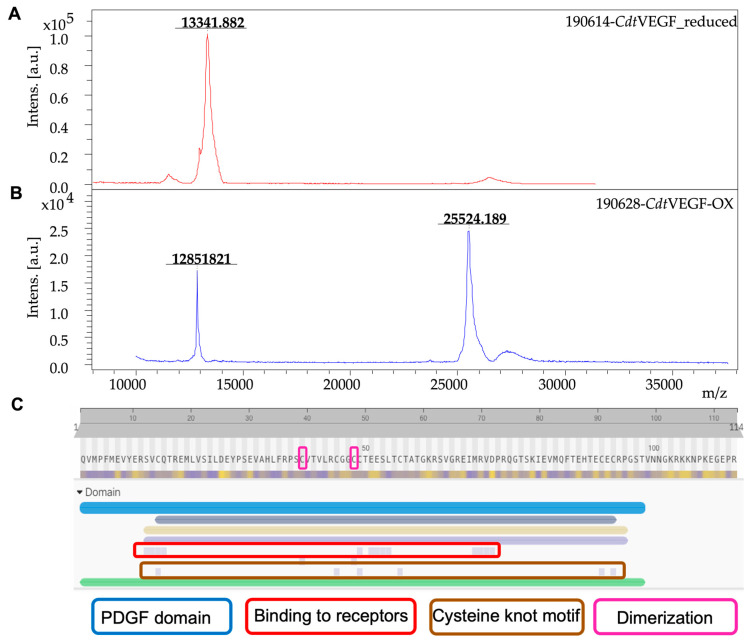
Mass spectrum and structural domains of *Cdt*VEGF. (**A**) MALDI-TOF (positive linear mode) of reduced/alkylated *Cdt*VEGF using DHB matrix; (**B**) oxidized *Cdt*VEGF; (**C**) PDGF domain (blue); binding to receptors (red); cysteine-knot motif (brown); and cysteines 39 and 48 (pink) responsible for molecule dimerization.

Mass spectrometry analysis (Figure 2A,B) revealed that *Cdt*VEGF reaches maximum elution at peak Q19-2 with a molecular mass of 13.3 Da as a monomer (reduced with DTT and alkylated with iodoacetamide), and a molecular mass of 25.5 Da as a dimer, as also demonstrated in the electrophoresis profile. In silico analysis of *Cdt*VEGF’s primary structure (Figure 2C) revealed the presence of a PDGF domain, a binding-to-receptors region, a cysteine-knot motif, and cysteine residues responsible for molecule dimerization (Cys39 and Cys48). Following the characterization of the *Cdt*VEGF primary structure, in MS/MS analyses after trypsin digestion of the samples, 36,956 spectra generating 3169 peptides were obtained. With these results, the primary sequence was obtained with 114 amino acid residues, which could not be completed since we used only one protease to determine it and the presented sequence ended in Arg residue (cleavage site for trypsin). The PMF provided a sequence coverage that encompassed nearly 83% of the proposed sequence. Finally, the obtained sequence is similar to other svVEGFs, with sequence identity between 56 and 95% and presenting the vascular homology domain a (VHD), a homology domain shared among svVEGFs. The protein sequence data reported in this paper will appear in the UniProt Knowledgebase under the accession number C0HM96.

**Figure 3:**
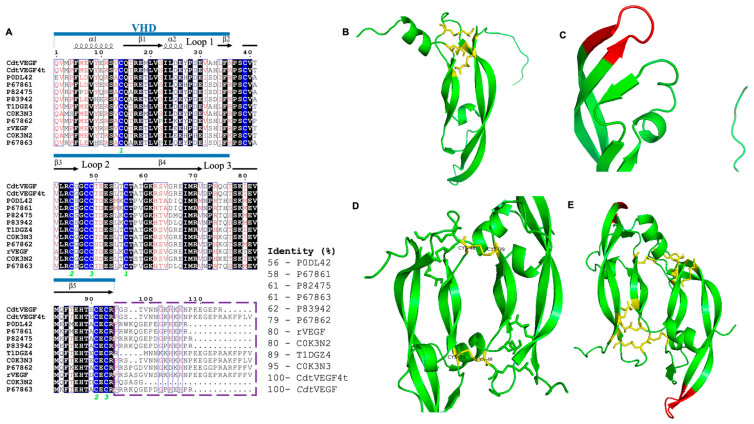
Sequence alignment of *Cdt*VEGF with SvVEGFs and molecular modelling. (**A**) CdtVEGF4 (*C. d. terrificus* transcriptome), *Daboia russelli siamensis* (P0DL42), *Daboia russelli russelli* (P67861), *Macrovipera lebetina* (P82475), *Vipera aspis aspis* (P83942), *Crotalus horridus* (T1DGZ4), *Crotalus atrox* (C0k3N3), *Protobothrops flavoviridis* (P67862), *Bothrops insularis* (rVEGF-Q90X24), *Agkistrodon piscivorus piscivorus* (C0k3N2), and *Vipera ammodytes ammodytes* (P67863). Red highlighting denotes conserved residues, while black highlighting signifies highly conserved residues. Cysteine residues are depicted in blue, with green numbers denoting disulfide bridges below them. Residues with significant similarity are enclosed in a blue box. β-sheets are represented by arrows, while α-helices are depicted as spirals to illustrate the secondary structures. Purple dotted box: C-terminal region responsible for binding to coreceptors. In blue: VHD. (**B**) monomeric form of *Cdt*VEGF with intrachain disulfide bonds highlighted in yellow; (**C**) monomeric form of *Cdt*VEGF with loop 3 region highlighted in red; (**D**) dimeric form of *Cdt*VEGF with interchain disulfide bond highlighted in yellow; (**E**) dimeric form of *Cdt*VEGF with intra- and interchain disulfide bonds and loop 3 region. Yellow shading is utilized to illustrate the presence of disulfide bonds, and loop 3 is highlighted in red. The alignment was generated using the ClustalOmega server, while ESPript was employed for the design [35]. Modeller and Swiss-Model were used to estimate the 3D structure of the molecule using the VEGF from *Vipera aspis* aspis (PDB: 1WQ8) as a template.

The elucidated sequence and its correlation among svVEGFs are shown in Figure 3A, and the PMF and spectra of MS/MS analysis are shown in the Supplementary Material (Figures S1–S3). Additionally, with the *Cdt*VEGF primary sequence, it was possible to predict/model its secondary and tertiary structures. According to HHpred, the structural model presented 67% of the identity and a probability of 99.88% with Vammin, which was chosen as the best template for building the structural model. We obtained *Cdt*VEGF in its monomeric structure using the Modeller program and in its dimeric structure using Swiss-Model (Figure 3B–E).

**Figure 4:**
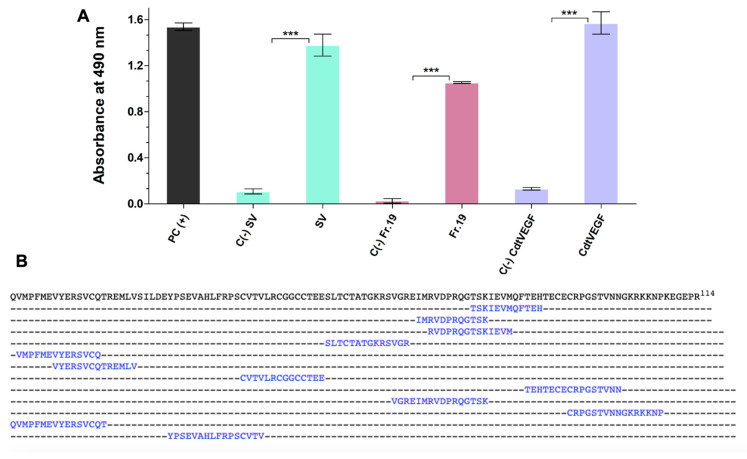
ELISA-based assay for *Cdt*VEGF recognition by anticrotalid antivenom and prediction of the recognized epitopes. (**A**) Samples (*Cdt*VEGF, fraction 19, and *C. d. terrificus* venom—2 μg each) were incubated with commercial anticrotalid antivenom from Instituto Butantan. Data are presented as mean ± SEM analyzed by one-way ANOVA followed by Tukey’s multiple comparison test (quadruplicate assay). PC(+): refers to wells coated with nonimmunized horse serum (1:50); SV, Fr.19, *Cdt*VEGF: samples were subjected to incubation with commercially available anticrotalid antivenom (1:100); C(−) of referenced samples incubated with commercially available anticrotalid antivenom (1:100).*** *p* < 0.0001 when compared to its negative control. (**B**) The *Cdt*VEGF sequence was examined, and the predicted epitopes, as identified by the ABCpred Server tool, are visually indicated in blue.

In a distinct assay, *Cdt*VEGF was subjected to incubation with a commercially available crotalid antivenom with the aim to evaluate its capability to bind and recognize this specific protein. The obtained results substantiated the ability of the Brazilian crotalid antivenom to effectively recognize the protein (Figure 4A). This outcome was in line with the in silico prediction, which anticipated the presence of 12 potential binding epitopes of the protein that have the potential to be recognized by B cells, thereby eliciting an immune response and prompting the production of specific antibodies (Figure 4B).

**Figure 5:**
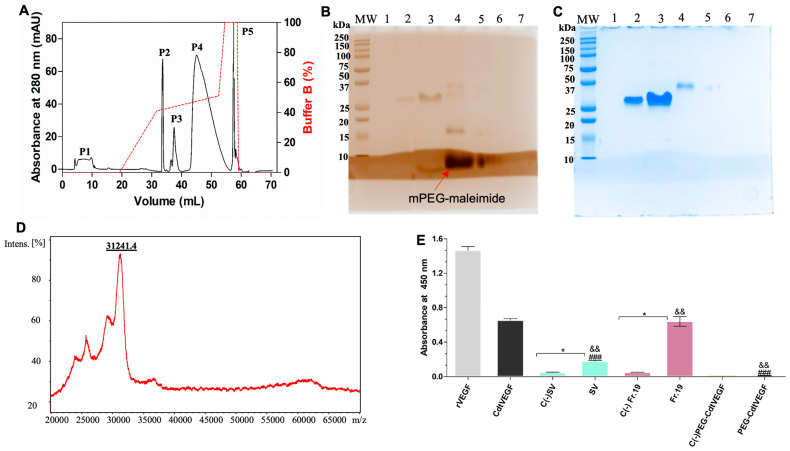
Chemical modification of *Cdt*VEGF by PEGylation and recognition by anti-VEGF-F ELISA assay. (**A**) PEG-*Cdt*VEGF chromatographed on a C4 analytical column. The red dotted line represents the gradient of solution B (%). (**B**,**C**) An amount of 15% SDS-PAGE of peaks P1→P5. Lane 1: peak 2; lane 2: native *Cdt*VEGF; lane 3: peak 3; lane 4: peak 4; lane 5: peak 5; lanes 6 and 7: empty. MW: molecular weight marker. The gel was first stained with barium-iodide solution and afterwards stained with Coomassie Brilliant Blue G-250. (**D**) Mass spectra of P4 analyzed by MALDI-TOF (positive linear mode) with sinapinic acid (SA) as the matrix. (**E**) The referenced samples (0.5 μg each) were incubated with anti-VEGF-F commercial antibody. Positive controls: wells coated with commercial rVEGF-F and native *Cdt*VEGF. C(−) SV, C(−) Fr.19, and C(−) PEG-*Cdt*VEGF: mentioned samples incubated with nonimmunized rabbit serum diluted 1:50. Absorbance was measured at 450 nm. Data are presented as mean ± SEM analyzed by one-way ANOVA followed by Tukey’s multiple comparison test (triplicate assay). * *p* < 0.01 when compared to negative control (−); ### *p* < 0.0001 when compared to positive control (rVEGF); and && *p* < 0.001 when compared to positive control (native *Cdt*VEGF).

After its structural characterization, *Cdt*VEGF was submitted to PEGylation with 5 kDa mPEG-maleimide. With an analytical reversed-phase C4 column, we accomplished the purification process of the PEGylated *Cdt*VEGF (PEG-*Cdt*VEGF) and obtained five chromatographic fractions (Figure 5A). The 15% SDS-PAGE demonstrated that PEG-*Cdt*VEGF is eluted at fraction P4 (Figure 5B,C), having an approximate molecular mass of 30 kDa, which was further confirmed as 31,241 Da through mass spectrometry of fraction P4 (Figure 5D). Also, we can observe that the mPEG-maleimide band could only be detected when the gel was stained with barium-iodide solution (Figure 5B). The PEGylation reaction of *Cdt*VEGF resulted in a yield of 76.32% (Table S1).

To assess the antibody recognition of PEG-*Cdt*VEGF, an ELISA assay was performed using anti-VEGF-F antibodies. The results revealed that anti-VEGF-F was able to recognize VEGF in a pool of *Cdt* venom and fraction 19 but did not recognize the PEG-*Cdt*VEGF (Figure 5E).

**Figure 6:**
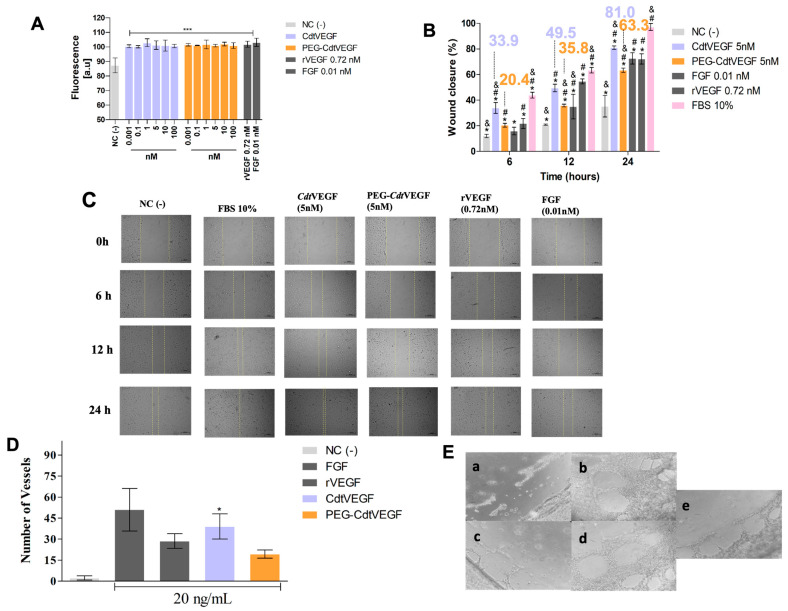
Functional in vitro assays. (**A**) Effect of native *Cdt*VEGF and PEG-*Cdt*VEGF on metabolic activity of HUVEC cells. HUVEC cells were incubated with different concentrations of native *Cdt*VEGF and PEG-*Cdt*VEGF (0.01–100 nM) in RPMI medium. *** *p* < 0.0001 when compared with the negative control (cells incubated with saline solution), and the presented values are the mean ± SEM (*n* = 3). (**B**,**C**) Evaluation of migration of HUVEC cells after different times (0–24 h) of treatment with native *Cdt*VEGF and PEG-*Cdt*VEGF (5 nM). NC(−) negative control (culture media + cells). The dashed yellow lines delimit the region of wound closure. Data (*n* = 3) are presented as mean ± SEM. * *p* < 0.01 when compared to the positive control (FBS 10%); # *p* < 0.01 when compared to the negative control; and & *p* < 0.01 when compared to rVEGF. (**D**,**E**) Angiogenic activity: (**D**) ability of *Cdt*VEGF and PEG-*Cdt*VEGF to induce HUVEC cells to form vessels; and (**E**) indicative photography of the vessels: (**a**) culture media (NC(−)); (**b**) culture media + FGF; (**c**) culture media + rVEGF; (**d**) culture media + *Cdt*VEGF; and (**e**) culture media + PEG-*Cdt*VEGF. * *p* < 0.01 when compared to NC(−). The assay was performed in triplicate and the results express the media ± SEM, which were analyzed by one-way ANOVA followed by Dunnett’s post hoc test.

In the in vitro functional assays, both *Cdt*VEGF and PEG-*Cdt*VEGF enhanced the metabolic activity of HUVEC cells in all tested concentrations (Figure 6A). Regarding the cellular migration of HUVEC cells towards a wound (scratch assay), the native and PEG-*Cdt*VEGF (5 nM) induced wound closure after 12 h of 49.5% and 35.8%, respectively (Figure 6B,C), demonstrating that the effects observed in the presence of both forms of *Cdt*VEGF were statistically significant when compared to the negative control. The angiogenic activity of *Cdt*VEGF (20 ng/mL, 0.78 nM) was revealed by its capability to prompt HUVEC cells to develop vessels on the in vitro assay through tube formation on Matrigel^®^. The PEGylated form also induces HUVEC cells to form vessels, but this effect was not statistically significant when compared to the negative control (Figure 6D,E).

**Figure 7:**
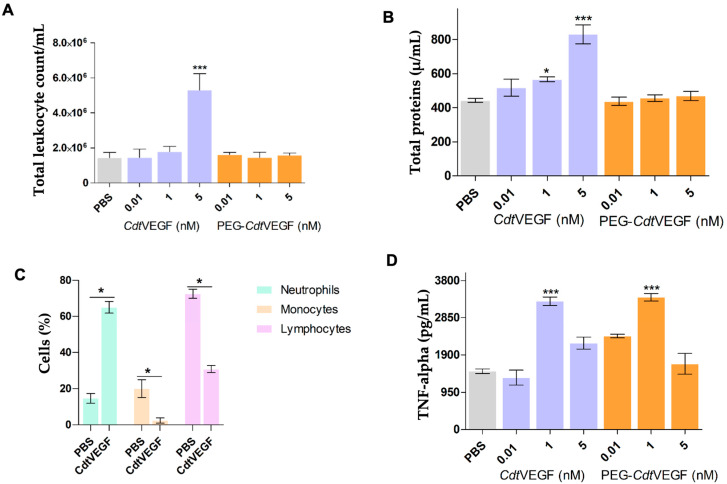
In vivo assay. Induction of leukocyte recruitment and vascular permeability analysis by native *Cdt*VEGF and PEG-*Cdt*VEGF. (**A**) The Neubauer chamber was utilized to quantify the total leukocyte count in the peritoneal cavity subsequent to the injection of PBS (negative control) or varying doses of *Cdt*VEGF and PEG-*Cdt*VEGF (0.01, 1, and 5 nM in 200 µL of PBS). (**B**) Total protein quantification was determined in the supernatant of the peritoneal exudates of animals euthanized 24 h after injection of PBS (NC) or different doses of *Cdt*VEGF and PEG-*Cdt*VEGF. (**C**) The assessment of leukocyte differentiation into neutrophils and mononuclear cells was conducted using an optical microscope. (**D**) Evaluation of cytokine TNF-α production. Results expressed as mean ± SEM (*n* = 5). *** p < 0.0001 when compared to negative control (PBS). * *p* < 0.01.

To evaluate the influence of *Cdt*VEGF and PEG-*Cdt*VEGF on leukocyte recruitment and vascular permeability, in vivo assays were performed. Native *Cdt*VEGF induced an increase in total leukocyte recruitment to the peritoneal cavity of male C57Bl/6 mice (Figure 7A). Since an increase in protein quantification of the peritoneal exudates was observed, it can be concluded that vascular permeability was also increased. Both effects were dose-dependent (Figure 7B) and compared to the control group (PBS) were statistically significant at 1 and 5 nM. Nevertheless, the administration of PEG-*Cdt*VEGF did not result in any statistically significant effect. In the differential cell count, *Cdt*VEGF induced an increase in the influx of neutrophil but it reduced lymphocytes and monocyte influx to the peritoneal exudates (Figure 7C). Regarding the cytokine measurement, 1 nM *Cdt*VEGF showed the most statistically significant increase in TNF-α in peritoneal exudates when compared to the control group, and PEG-*Cdt*VEGF (1 nM) also increased the levels of this cytokine (Figure 7D).

## 3. Discussion

Prior research has provided valuable insights into the identification of VEGF-like proteins derived from various snake venoms, showcasing their diverse functional roles beyond angiogenesis. These roles include influencing vascular permeability, promoting endothelial cell proliferation, and facilitating monocyte migration [18,26,28,31,36,37]. The characterization of proteomes and transcriptomes of numerous snake venoms has further highlighted the prominence of studies on snake venom VEGF-like proteins (svVEGFs) due to their presence across multiple snake families [16,34,38,39,40,41]. While their involvement in inducing vascular permeability during envenoming has been reported, further investigations are necessary to fully comprehend their functionalities.

In this study, we successfully isolated *Cdt*VEGF through a two-step chromatographic process. The initial step involved reversed-phase chromatography on a semipreparative C18 column, which played a critical role in obtaining fraction 19. The presence of svVEGF in this fraction was confirmed using anti-VEGF-F antibody. Subsequently, anion exchange chromatography was employed to purify the proteins based on their charge. The reported isoelectric points (pIs) of svVEGFs range from 7.8 to 8.7, as documented in previous studies [26,27,31,42]. In our investigation, at a pH of 7.8, *Cdt*VEGF demonstrated a moderate binding capacity to the anionic resin employed in the exchange chromatography, leading to its elution at an intermediate point along the gradient. This step was crucial for separating *Cdt*VEGF from the major contaminant of fraction 19, PLA_2_ [16,28,41], which possesses a more acidic isoelectric point and required elution with high concentrations of ammonium bicarbonate (AMBIC) buffer. Following *Cdt*VEGF isolation, its presence in the soluble venom is in relatively low abundance (approximately 2%). This finding aligns with previous studies that described svVEGFs as minor components of snake venoms [43].

Our analysis revealed that *Cdt*VEGF exhibited migration as a dimeric protein during electrophoresis, consistent with findings reported for svVEGFs from various snake species, including *Vipera lebetina* [36], *Gloydius tsushimaensis* [31], *Protobothrops mucrosquamatus* [44], *Protobothrops jerdonii* [26], and *Crotalus durissus collilineatus* [28]. Furthermore, mass spectrometry analysis of *Cdt*VEGF using MALDI-TOF was performed, and the results demonstrated that *Cdt*VEGF (after being reduced and alkylated) exhibited a molecular mass of 13.3 Da and 25.5 Da as a dimer in oxidized form. These findings align with previously reported molecular masses of svVEGFs from *Protobothrops jerdonii* (29 kDa) [26], *Gloydius tsushimaensis* (26 kDa) [31], and *Crotalus durissus collilineatus* [28].

Concerning the structural characterization of *Cdt*VEGF, we performed mass spectrometry analysis using digestion with trypsin, given that a substantial number of svVEGFs possess their amino terminal residue blocked by pyroglutamic acid [28,36,42].

Upon conducting spectra analysis using PeaksStudio XPro software and referring to databases encompassing comprehensive amino acid sequences of proteins from snakes, as well as amino acid sequences of proteins from the *Crotalus* genus, we successfully identified peptides exclusively attributed to svVEGFs. Hence, we conducted supplementary analysis by employing a more stringent database that exclusively encompassed amino acid sequences of svVEGFs from the *Crotalus* genus, aiming to enhance our identification process. By integrating the findings derived from both LC-MS/MS and PMF analysis, the determined sequence of *Cdt*VEGF exhibits remarkable similarity to other svVEGFs and shares a striking 95% identity with CdtVEGF4, previously identified in *Cdt* transcriptome analysis. In silico analysis of the *Cdt*VEGF primary structure revealed the presence of a PDGF domain, which confers to VEGFs the capability to promote the proliferation of endothelial cells.

According to the findings of Yamazaki et al. (2009), a moderate scope of accelerated evolutionary modification has been observed in the coding region of VHDs (vascular homology domains) within svVEGFs. This accelerated evolution gives rise to a diverse range of functional variations, particularly in crucial regions such as the putative receptor-binding loops 1 and 3, as well as the C-terminal putative coreceptor-binding region. In contrast, the sequences of mammalian VEGFs exhibit a high degree of conservation in these regions [27]. The VHD domain comprises 92–96 amino acids, displaying from 29 to 64% identity across the VEGF family. It is noteworthy that the eight cysteine residues and loops 1, 2, and 3 play significant roles in the VHD domain [10].

In this study, we used the template of 1WQ8 to model *Cdt*VEGF in both monomeric and dimeric forms (Figure 3A). Remarkably, both forms exhibit the same number of disulfide bonds, with three intrachain and two interchain bonds, as well as a conserved loop 3 region, consistent with the model previously constructed by Suto et al. [19]. Notably, within the loop 3 region, *Cdt*VEGF possesses a glutamine (Q) residue at position 75 in contrast to Vammin (P67863), which features a threonine (T) at the same position. Diverse conformations of the loop 3 region have been observed in svVEGFs, primarily attributed to the presence or absence of a threonine residue at position 75 (Thr75), and the variability in this specific amino acid composition plays a crucial role in receptor binding, contributing to the diverse functional activities exhibited by svVEGFs [19].

Crotalid antivenom demonstrated the ability to recognize *Cdt*VEGF, indicating its immunogenicity in animals, particularly horses, despite constituting only a minor portion of snake venoms [43]. According to the authors’ suggestions, epitopes that exhibit optimal immunogenicity are recommended to possess a size larger than 10 residues but should not exceed 20 residues [45,46]. Indeed, the epitope prediction unveiled that *Cdt*VEGF encompasses twelve epitopes with lengths that could potentially be recognized by the immune system.

Nevertheless, various approaches can be employed to mitigate the immunoreactivity of molecules obtained from animals, such as utilizing alternative delivery routes or implementing chemical modifications like PEGylation [11]. Prior investigations have presented convincing findings demonstrating that Collinein-1, a thrombin-like serine protease extracted from *C. durissus collilineatus*, can undergo successful site-specific PEGylation, leading to a substantial augmentation of its thrombin-like activity in comparison to its native state [47]. Furthermore, a recent investigation involving the same protein revealed that the PEGylated variant displayed nonimmunogenic characteristics when administered to mice [48]. In light of these findings, PEGylation is considered a promising technique in this context [49,50].

Assuming that *Cdt*VEGF is immunogenic on the basis of the ELISA assays and considering its potential application as a therapeutic compound, we proceeded with the chemical modification of this protein through PEGylation. To purify PEG-*Cdt*VEGF, reversed-phase chromatography was employed, and the fractions obtained were analyzed through SDS-PAGE stained with barium-iodide solution and afterwards with Coomassie Brilliant blue G-250. Our results showed that when stained with barium-iodide solution, PEGylated CdtVEGF, mPEG-maleimide, and non-PEGylated proteins appeared on the gel. By the principle of the barium-iodide method for staining PEG molecules, according to Kurfürst, M.M., 1992 [51], the coloration can be more intense for PEGylated proteins, it can detect PEG molecules free or bound to proteins, and excess of PEG molecules can be identified as well. The literature data also demonstrate non-PEGylated proteins stained with barium-iodide solution, including the molecular weight markers [52,53]. The application of PEGylation could potentially impact the protein’s structure–function relationship during the purification process. PEGylation can reshape the molecular landscape, sculpting its conformation, harmonizing electrostatic bonds, and modulating hydrophobic tendencies. As a result, these modifications may lead to a reduced biological activity of the PEGylated protein compared to its native form. The decrease in activity can be attributed to the steric hindrance effect caused by the highly hydrophilic nature of the PEG chains. This effect has the potential to interfere with the binding of the PEGylated protein to specific receptors or interfere with protein–protein interactions [54].

Our data indicate that PEGylation was successful, and the in vitro functional assays demonstrated that PEG-*Cdt*VEGF induced metabolic activity on HUVEC cells comparable to the native *Cdt*VEGF, as determined by the resazurin assay. The evaluation of cellular migration is of significant importance in the study of wound healing processes. This dynamic phenomenon can be categorized into five distinct stages and presents a crucial role in restoring the functional integrity of the skin barrier involving the coordinated actions of various cell types [55]. In vitro assays for wound healing are based on the fundamental concept of intentionally creating a disrupted cell monolayer, resulting in the formation of an empty cell region that can be utilized to investigate migration and repair processes following treatment with the compound of interest [56].

For svVEGFs, to the best of our knowledge, the induction of migration of endothelial cells through scratch assay has not been reported so far. In this manner, we could establish a pioneering determination of this role for *Cdt*VEGF and PEG-*Cdt*VEGF. Our data revealed that both forms of the protein were able to induce time-dependent migration of HUVECs cells, with more expressive values of wound closure within 12 and 24 h.

VEGFs constitute a pivotal family of proteins with significant relevance in stimulating neovascularization in endothelial cells, thereby fostering the formation of novel blood vessels [25]. For svVEGFs, this function is poorly explored since there are, so far, few studies reporting their ability to form new vessels [28,44,57]. One of those is ICPP, a svVEGF from *Macrovipera lebetina* snake venom, which could induce neovascularization on HUVEC cells in a 10 ng/mL concentration, while human VEGF promoted the formation of new vessels with a 30 ng/mL concentration [57]. Also, *Cdc*VEGFs, from *C. d. collilineatus* snake venom, were shown to induce formation of new vessels in a tube formation assay when tested at 20 ng/mL concentration, surpassing the potency of human FGF used at the same concentration [28]. On the other hand, regarding angiogenesis, a svVEGF (Pm-VEGF) less potent than human VEGFs was reported. Pm-VEGF, from *Protobothrops mucrosquamatus* snake venom, could only induce neovascularization in the chick chorioallantoic membrane assay at a concentration ten times higher than that of human VEGF [44].

In our study, we also investigated the capacity to stimulate angiogenesis in vitro, and our results demonstrated that *Cdt*VEGF (20 ng/mL, 0.78 nM) was able to induce new vessels formation in HUVEC cells culture when compared to the negative control. Nevertheless, PEG-*Cdt*VEGF (20 ng/mL, 0.64 nM) induced less formation of vessels than the native form. These results provide further evidence that PEGylation has the capacity to impact the activities of the molecule.

The mPEG-maleimide has preference for free cysteine residues, and a second preferential pathway is the binding to δ-amine of lysine [58]. Taking into consideration that *Cdt*VEGF contains a glutamine (Q) residue at the N-terminal position, which has the potential to undergo cyclization and form pyroglutamic acid, we did not consider that the reaction occurred at an N-terminal residue, even though at pH 7.4, the reactivity of the amino group at the N-terminus would be higher. Therefore, we formulated the hypothesis that mPEG-maleimide underwent conjugation with the lysine amino acids that exist within the structure of *Cdt*VEGF, although more experimental analysis would be necessary to confirm it. Subsequently, when we subjected PEG-*Cdt*VEGF to analysis employing antibody specific for svVEGFs, it failed to exhibit recognition of PEG-*Cdt*VEGF. This observation prompted us to consider the possibility that the epitopes present in the *Cdt*VEGF molecule might have been masked by the mPEG-maleimide molecule, as there are lysine residues within the epitopes.

The mobilization of leukocytes from the circulatory system to target tissues assumes a pivotal function in both the inflammatory cascade and immune response, serving as an indispensable biological mechanism. As a result, the extravasation of leukocytes is directly involved with the vascular endothelium and activation of molecules derived from vessel walls that are closely associated with vascular permeability [59], and VEGFs can affect the leucocyte interaction with wall vessels [60,61]. Regarding svVEGFs, Vammin has already demonstrated its potential to induce vascular permeability, being fivefold more potent than human VEGF, evoking distinct changes in vascular ultrastructure with preference on the formation of fenestrae, forming pores in the vascular endothelium, and facilitating the leakage of other toxins and proteins [27]. However, the correlation of svVEGFs with inflammation or leukocyte recruitment was not yet elucidated, and it is crucial to expand the understanding of their role during snakebite envenoming.

*Cdt*VEGF and PEG-*Cdt*VEGF were investigated for their ability to recruit leukocytes to the peritoneal cavity and induce vascular permeability in an animal model using C57Bl/6 male mice. The native *Cdt*VEGF demonstrated a significant capacity to induce leukocyte recruitment, with the highest concentration (5 nM) resulting in the most pronounced recruitment response. Interestingly, when tested at equivalent concentrations, PEG-*Cdt*VEGF did not induce any recruitment, indicating that the process of PEGylation may mitigate the potential immune response initiated by *CdtV*EGF upon inoculation. Leukocytes, including neutrophils, play a crucial role as key components of the innate immune response. They act as the first line of defense in regulating the inflammatory response that occurs as a result of tissue damage, which is a characteristic feature of snakebites [62].

Performing the differential leukocyte count after administering 5 nM *CdtV*EGF demonstrated significant changes, clearly indicating an influx of neutrophils. Typically, under normal conditions, C57Bl/6 mice display a neutrophil percentage ranging from 8 to 20% [63]. However, our findings revealed that the group of animals inoculated with 5 nM *Cdt*VEGF exhibited a remarkable neutrophil percentage of approximately 60%, thereby confirming the inflammatory response induced by *Cdt*VEGF. In addition to neutrophils, *Cdt*VEGF also induced a minor influx of lymphocytes. However, it is worth noting that the control group exhibited a high percentage of lymphocytes, which aligns with the existing literature data indicating that C57Bl/6 male mice typically have approximately 70–89% lymphocytes. Furthermore, under normal circumstances, these mice demonstrate low levels of monocytes (0–4%), which further supports our findings observed in the control group [63].

To evaluate the vascular permeability that *Cdt*VEGF could induce, total protein quantification was accomplished on the peritoneal exudates, revealing a dose-dependent increase in proteins induced by native *Cdt*VEGF. This increase in protein levels was correlated with the elevation in vascular permeability, facilitating the extravasation of proteins into the peritoneal cavity. In contrast, PEG-*Cdt*VEGF did not induce the observed increase in protein levels that was evident in the presence of *Cdt*VEGF.

The infiltration of leukocytes into the peritoneal cavity accompanied by a concurrent increase in total protein levels triggered by *Cdt*VEGF offer compelling evidence of its capacity to incite local inflammation—a characteristic that remains unexplored within this particular protein class. The induction of vascular permeability from *Cdt*VEGF corroborates reports of other svVEGFs that also led to an increase when tested in animal models at low concentrations (10 ng/mL, 0.5 nM) [18,26,31,57,64]. In another study, svVEGF IC2 from *M. lebetina* snake venom, when tested on C57Bl/6 mice, induced higher vascular permeability than when tested on Swiss lineage [42].

Cytokines play a pivotal role in facilitating the recruitment of leukocytes by promoting the transmigration of adherent leukocytes across the endothelium and extracellular matrix [59]. These signaling molecules form an intricate network that guides leukocytes to specific sites, underscoring the vital role of cytokine-mediated regulation in orchestrating the inflammatory response and preventing tissue damage [65]. TNF-α is an important pro-inflammatory cytokine that promotes expression of cell adhesion molecules and activation of B and T cells, with a crucial contribution to leukocyte recruitment and inflammation [66].

In our study, when we measured TNF-α levels in peritoneal exudates, native *Cdt*VEGF exhibited the ability to enhance cytokine production, with 1 nM concentration showing the most significant effect compared to the control group treated with PBS. Although PEG-*Cdt*VEGF did not display any activity in terms of leukocyte recruitment and vascular permeability, it maintained its activity in inducing soluble mediators, as demonstrated by the production of TNF-α. Importantly, there is currently no evidence suggesting that PEGylation of proteins affects the distribution of circulating cytokines [67].

## 4. Conclusions

This work represents the pioneering isolation and full structural/functional characterization of the vascular endothelial growth factor from Brazilian rattlesnake venom (named *Cdt*VEGF). It also reports the first-time PEGylation of a svVEGF, with the resulting PEG-*Cdt*VEGF exhibiting in vitro activities comparable to native *Cdt*VEGF. Specifically, PEG-*Cdt*VEGF retains its capacity to facilitate the proliferation, migration, and formation of tubular structures in HUVEC cells. Moreover, PEG-*Cdt*VEGF exhibits reduced inflammatory activity in C57Bl/6 mice, thereby creating new opportunities and perspectives for future investigations targeting the understanding of their in vivo behavior. These results also bring to light the potential role of *Cdt*VEGF during envenoming due to its inflammatory response, leading to an elevation of total proteins on peritoneal exudates with the consequent increase in vascular permeability. The potential implications of *Cdt*VEGF as a valuable research and therapeutic tool are evident, given its multifunctional properties that can be harnessed to manipulate various natural and disordered processes, including angiogenesis, ischemic disorders, and wound healing facilitation.

## 5. Materials and Methods

### 5.1. Venoms, Cells, and Animals

*C. d.* terrificus snake venoms were obtained from fully grown snakes and housed at the Serpentarium of the University of São Paulo, located within the Ribeirão Preto Medical School. The Serpentarium holds accreditation from the Brazilian Institute of Environment and Renewable Natural Resources, registered under the number 1506748.

Male C57Bl/6 mice weighing 18–20 g were supplied by the animal facilities located at the University of São Paulo, Ribeirão Preto Campus, São Paulo. All experiments were performed in accordance with the Ethical Principles in Animal Experimentation and approved by the Animal Care and Use Committee of the School of Pharmaceutical Sciences of Ribeirão Preto—USP (CEUA-FCFRP), under the protocol number 21.1.611.60.6.

HUVEC (human umbilical vein endothelial cells) were sourced from the American Type Culture Collection (ATCC, Cat. No. CRL-4053). The cells were cultured in RPMI 1640 medium (Roswell Park Memorial Institute 1640 Medium; Gibco, Carlsbad, CA, USA) supplemented with 10% Bovine Fetal Serum (FBS; Gibco, Carlsbad, CA, USA), 1% antibiotic/antimycotic mix (10,000 U/mL penicillin, 10 mg/mL streptomycin, and 25 μg/mL amphotericin B; Gibco, Carlsbad, CA, USA), and 0.024% sodium bicarbonate (Sigma-Aldrich, St. Louis, MO, USA). The cells were maintained in a humidified incubator at 37 °C, 5% CO_2_, and 95% relative humidity.

### 5.2. CdtVEGF Isolation

Desiccated venom (50 mg) was fractionated on a C18 column (10 × 250 mm, 5 µm, 300 A semipreparative column) according to Calvete and colleagues [68] with modifications, and the elution of protein was monitored at 214 nm. According to the ELISA assay, fractions that indicated the presence of VEGF were fractionated through anion exchange chromatography on a HiTrap^®^ Q XL Sepharose Fast Flow column (5 mL, 0.7 × 2.5 cm, GE Healthcare, Uppsala, Sweden), which was previously equilibrated with 10 mM ammonium bicarbonate, pH 7.8, as described by Ferreira, I.G. et al. [28], and elution of the proteins was monitored at 280 nm. Following that, the lyophilized subfractions were subjected to molecular exclusion column chromatography using a HiTrap™ Desalting column (1.6 × 2.5 cm, 5 mL, GE Healthcare, Uppsala, Sweden) with an isocratic gradient of 0.1% TFA (trifluoroacetic acid), monitored at 280 nm. The fractions were lyophilized and stored at −20 °C until use. All the purification steps were carried out utilizing an Äkta Purifier UPC10 system, a Fast Protein Liquid Chromatography platform (GE Healthcare, Uppsala, Sweden). Protein quantification was performed according to the Scopes method [38]. After its isolation, *Cdt*VEGF was chromatographed using a heparin column (5 mL, 0.7 × 2.5 cm, HiTrap™ Heparin HP, GE Healthcare^®^, Little Chalfont, UK) to analyze its affinity towards heparin. The column was connected to an FPLC system with 2 mL/min flow and was pre-equilibrated with Tris-HCl 25 mM buffer, pH 8.9, and the protein was eluted using a segmented gradient of NaCl (0–0.4 M) with absorbance at 280 nm.

### 5.3. Recognition of CdtVEGF and PEG-CdtVEGF Using Enzyme-Linked Immunosorbent Assay (ELISA)

To assess the recognition of *Cdt*VEGF and PEG-*Cdt*VEGF by anti-VEGF-F antibodies, an ELISA assay was conducted. A 96-well microplate (Costar, Corning Incorporated) was coated with 0.5 µg of samples (*Cdt*VEGF, PEG-*Cdt*VEGF, or a pool of Cdt venom) diluted in 0.05 M carbonate/bicarbonate buffer, pH 9.6 (100 µL/well). As a negative control, the wells coated with the referenced samples were incubated with nonimmunized rabbit serum diluted 1:100 in 1% MPBS. The assay was carried out in triplicate, following methodology described by Ferreira et al. [28].

### 5.4. SDS-PAGE

An amount of 15% SDS-PAGE were prepared according to the Laemmli method [69]. Venom fractions containing *Cdt*VEGF and PEGylated fractions (5 μg) were analyzed under reducing and nonreducing conditions. Low molecular mass (97.0–14.4 kDa, 17-0446-01, GE Healthcare, Uppsala, Sweden) and wide molecular mass (250–10 kDa, 161-0374, BioRad, Brazil) markers were used. For the PEGylated protein, gels were stained with barium-iodide solution following the Skoog method [70] and with Coomassie Brilliant Blue G-250 (Sigma-Aldrich, St. Louis, MO, USA) for total proteins.

### 5.5. Reactivity of Crotalid Antivenom towards CdtVEGF and Prediction of Potential Epitopes within the Molecule

The assay was performed following methodology from Oliveira et al., 2021 [71]. For epitope prediction, an ABCpred Server was used, with a breakpoint of 0.8 and a window length of 12 to 16 residues [72].

### 5.6. Molecular Mass of CdtVEGF

A quantity of 5 μg of *Cdt*VEGF was subjected to reduction using 10 mM dithiothreitol (DTT) and incubated at 56 °C and 600 rpm for 40 min. Subsequently, alkylation was performed with 20 mM iodocetoamide (IAA) at room temperature (RT) for 30 min in the dark. After, the sample was desalted using a reversed-phase ZipTip^®^ C4 column (ref. ZTC04S096, Millipore, Burlington, MA, USA), and the average molecular mass of *Cdt*VEGF was determined by matrix-assisted laser desorption/ionization with a time-of-flight analyzer (MALDI-TOF, RapifleX, Bruker Corporation, Billerica, MA, USA), and FlexControl 4.0 software (Bruker Corporation, Billerica, MA, USA) was employed for data acquisition during the analysis. To obtain data, 10,000 laser shots were captured per spectrum, and the laser frequency was set at 500 Hz. The instrument was operated in linear positive mode, covering a mass range of 10–50 kDa, and RapifleX was calibrated using Protein Standard I (ref. 8206355, Bruker Corporation, Billerica, MA, USA). A saturated solution of 2,5-dihydrobenzoic acid (DHB) in a mixture of acetonitrile (ACN) and 0.1% trifluoroacetic acid (TFA) at a 1:1 ratio served as the matrix for the analysis. Data analysis was performed using FlexAnalysis 3.4 software (Bruker Corporation, Billerica, MA, USA). PEGylated *Cdt*VEGF (5 μg) was analyzed as described above, using sinapinic acid (SA) as the matrix (8201345, Bruker Corporation^®^, Billerica, MN, USA), prepared in ACN and 0.1% TFA at the ratio of 3:7.

### 5.7. Amino Acid Sequence Determination of CdtVEGF

*Cdt*VEGF (5 μg) was reduced/alkylated as described in Section 5.6. Afterward, a second reduction with 11 mM DTT at RT for 5 min in the dark was performed. The sample underwent digestion with trypsin (0.2 μg; ref. 90058, Thermo Fisher Scientific Inc., Waltham, MA, USA) at a ratio of 1:50 (trypsin:*Cdt*VEGF) and incubated at 37 °C and 600 rpm overnight. Subsequently, a second digestion was carried out with trypsin at a ratio of 1:100 (trypsin:*Cdt*VEGF), followed by the addition of 80% acetonitrile. The sample was further incubated at 37 °C and 600 rpm for 3 h. To halt the reaction, 10% *v*/*v* trifluoroacetic acid (TFA) was added, and the sample was desalted using a reversed-phase solid-phase extraction column. The protein’s peptide mass fingerprint (PMF) was obtained using the same MALDI-TOF equipment mentioned in Section 5.6. However, for this analysis, the instrument was operated in reflectron positive mode and a mixture of peptides was used for calibration (peptide calibration standard, ref. 206195, Bruker Corporation, Billerica, MA, USA). A matrix solution of 10 mg/mL 2,5-dihydroxybenzoic acid (DHB) in a 1:1 ratio of MeCN (acetonitrile) and 0.1% TFA (trifluoroacetic acid) was prepared. Data analysis was performed using FlexAnalysis 3.4 and BioTools 3.2 software (Bruker Corporation, Billerica, MA, USA). The digested peptides were subjected to MS/MS fragmentation using the RapifleX instrument, controlled by FlexControl 4.0 software for data acquisition. MS/MS spectra were analyzed using FlexAnalysis 3.4 and Sequence Editor 3.2 software (Bruker Corporation, Billerica, MA, USA).

For LC-MS/MS, *Cdt*VEGF (10 μg) was reduced, alkylated, and digested with trypsin as described above, and the reaction was stopped with 0.5% TFA. The analysis was performed following the methodology of Oliveira, I.S et al. [71]. The MS/MS spectra were subjected to automated de novo sequencing using Peaks Studio X software for interpretation (Bioinformatics Solutions Inc., Waterloo, ON Canada) against databases extracted by our group. These databases were generated from the UniProt Knowledgebase (UniProtKB, https://uniprot.org, accessed on 3 May 2022) and comprised protein sequences found in snakes (274.615 sequences), Crotalus (12.118 sequences), and VEGF Crotalus (14 sequences) without signal peptide. Parent mass and fragment mass error tolerance were set to 5 ppm and 0.015 Da, respectively. As fixed modifications, carbamidomethylated cysteine was set and methionine oxidation and cyclization of pyroglutamic acid (Q or E) were regarded as variable modifications, while up to 3 missed cleavages per peptide were permitted during the analysis.

### 5.8. CdtVEGF Homology Model

Homology modelling was performed using the svVEGF Vammin, from *Vipera aspis aspis* snake venom (PBD ID: 1WQ8), as a template, chosen through a previous search by HHpred [73]. For the construction of the model, one structure was conducted using the MODELLER program [74] (http://toolkit.tuebingen.mpg.de/MODELLER), while the other structure was created using the Swiss-Model web server (http://swissmodel.expasy.org/) [75]. The analysis was conducted using the Procheck program [76] (http://swissmodel.expasy.org/workspace/index.php?func = tools_structureassessment1, accessed on 23 January 2023), and the molecular structures of *Cdt*VEGF were designed using PyMOL 1.7.4.4.

### 5.9. Bioconjugation of CdtVEGF with mPEG-Maleimide

The PEGylation and purification of PEG-*Cdt*VEGF was performed following the procedures described by Da-Silva-Freitas et al. [47] and Pinheiro-Junior et al. [48], with minor modifications, using 5 kDa maleimide-mPEG (Sigma Aldrich, DE, Darmstadt, Germany). The reaction process and the purity of the PEGylated proteins were evaluated with a 15% SDS-PAGE [39] under barium-iodide staining.

### 5.10. In Vitro Assays

#### 5.10.1. Angiogenesis

This assay was performed according to the methods of Grant et al. and Yeh et al. [77,78]. The effect of angiogenesis caused by both forms of *Cdt*VEGF (native and PEGylated) was analyzed by tube formation on Matrigel^®^. The cells (2 × 10^4^ cells/well) were pre-incubated for 30 min at 37 °C with: (i) culture medium (negative control); (ii) 20 ng/mL (1 nM) of recombinant FGF (fibroblast growth factor—13256029—Thermo Fisher Scientific Inc.), used as control; (iii) 20 ng/mL (0.72 nM) of recombinant svVEGF—rVEGF (AR31064PU-L, OriGene Technologies), also used as control; (iv) 20 ng/mL (0.78 nM) of *Cdt*VEGF; and (v) 20 ng/mL (0.68 nM) of PEG-*Cdt*VEGF. Following the incubation period, the cells were attached to a culture chamber coated with 50 μL of Matrigel (5.25 mg/mL, Corning Matrigel Matrix, Tewksbury) and maintained for 18 h. Subsequently, images were captured using an inverted microscope (Nikon^®^ Eclipse TS-100) equipped with a 20× objective lens. Five pictures were taken per well, including pictures of the center and four cardinal points. The total number of spherical structures formed by endothelial cells/vessel-like structures was then quantified. For statistical analysis, three wells were seeded per experimental condition.

#### 5.10.2. Metabolic Activity

The metabolic activity of HUVEC cells was performed using the resazurin reduction assay (Sigma-Aldrich, St. Louis, LO, USA), according to Page and Noel [79]. The fluorescence was analyzed on a Synergy™ HTX Multiplate Reader (Biotek^®^, Winooski, VT, USA), with an excitation of 530 nm and an emission of 590 nm. Cell metabolic activity was considered an indirect indicator of cell proliferation. Three distinct experiments were conducted and subjected to rigorous statistical analysis.

#### 5.10.3. Wound Healing Assay

*Cdt*VEGF and PEG-*Cdt*VEGF (5 nM each) were used, and the assay was performed according to Liang, Park, and Guan [80]. In our study, HUVEC cells (1.5 × 10^5^ cells/well) were cultured in a 12-well plate (Costar, Corning Incorporated, NY, USA) until reaching approximately 90% confluence. Subsequently, a sterile tip was used to create a small incision on the surface of the adherent cell monolayer. Afterward, the culture medium was refreshed to remove any cellular debris, and a fresh culture medium containing *Cdt*VEGF and PEG-*Cdt*VEGF was introduced. As negative controls, cells-containing medium was used. Three independent experiments were performed and statistically analyzed. Images of the wounds were recorded with a camera coupled to the inverted microscope (Zeiss-Primovert, Zeiss, Oberkochen, Germany) at different incubation time intervals (0, 6, 12, and 24 h) and the mean distance of the internal area was determined using Image J software 1.53i version (National Institutes of Health, Bethesda, MD, USA) according to the following equation:

$$Cell\;migration\;\left(\%\right)=\frac{\left(TA=0\;h\right)-\left(TA=24\;h\right)}{\left(TA=0\;h\right)}\times 100$$

where *TA* is the total area.

### 5.11. In Vivo Assays

#### 5.11.1. Evaluation of Leukocyte Influx into the Peritoneal Cavity

Male C57Bl/6 mice (*n* = 5) were intraperitoneally injected (i.p.) with *Cdt*VEGF or PEG-*Cdt*VEGF (0.01, 0.1, and 5 nM diluted in 200 μL of sterile PBS) and the controls received 200 μL of sterile PBS. After 24 h, the animals received via i.p. 60 mg/kg of ketamine (Dopalen, Agripands Brasil Ltd., Paulínia, SP, Brazil) and 8 mg/kg of xylazine (Rompun, Bayer Animal Health, Porto Alegre, RS, Brazil). Subsequently, 2 mL of PBS was gently introduced into the abdominal cavity and massaged for 1 min. Peritoneal fluid was obtained by inserting a needle attached to a syringe into the inguinal region, allowing for the collection of peritoneal cells. The total number of peritoneal cells was then quantified using Neubauer chambers in Turk’s solution. Differential counts of peritoneal leukocytes were conducted on cytospin preparations. The preparations were stained using a commercially available kit and subjected to the Romanowsky staining procedure (Panótico, Laborclin, Paraná, Brazil). After centrifugation (400× *g*, for 10 min, at 4 °C), the cell-free peritoneal fluid was used for the following dosages.

#### 5.11.2. Protein Quantification

The quantification of total proteins was performed on the cell-free peritoneal fluid obtained from the injected mice with *Cdt*VEGF or PEG-*Cdt*VEGF by Coomassie protein assay reagent (5000001, Bio-Rad, Rio de Janeiro, Brazil), according to the manufacturer’s instructions [81].

#### 5.11.3. Cytokine Measurement

The cell-free peritoneal fluid obtained from mice that were injected with either CdtVEGF or PEG-CdtVEGF was utilized to quantify TNF-α levels through an ELISA assay. This involved the use of specific antibodies (purified and biotinylated) and a cytokine standard, following the instructions provided by the manufacturer (Sigma-Aldrich, USA). Optical densities were measured at 405 nm using a microplate reader (Tecan-Sunrise, Männedorf, Switzerland). The cytokine levels for each sample were determined based on a standard curve generated with the appropriate recombinant cytokine, and the results were expressed in pg/mg of total protein. The assay had a sensitivity greater than 10 pg/mL.

### 5.12. Statistical Analysis

The results were expressed as mean values ± standard deviations (SD). Statistical significance of the results was evaluated using GraphPad Prism 5 software, using one-way analysis of variance (ANOVA) followed by Tukey’s or Dunnett’s post hoc test. Results were statistically significant as follows: *p* < 0.05; * *p* < 0.05; ** *p* < 0.01; *** *p* < 0.001; and *p* < 0.05.

## Figures and Tables

**Table 1:** *Cdt*VEGF recovery during purification steps.

Sample	Mass (mg)	Purification Steps	Recovery (%)
Crude venom	50.0	Supernatant	100
Fraction 19	3.1	Reversed phase	6.2
*Cdt*VEGF	1.0	Anion–ion exchange	2.0

## Data Availability

Not applicable.

## Supplementary Materials

The following supporting information can be downloaded at: https://www.mdpi.com/article/10.3390/toxins15080483/s1, Figure S1: Peptide mass fingerprint of *Cdt*VEGF digested with trypsin; Figure S2: Sequence coverage of *Cdt*VEGF determined by peptide mass fingerprint; Figure S3: Mass spectra of matching peptides; Table S1: Percentage of recovery from fractions obtained with PEGylation.
