# Supplementary material for: Beyond Angiogenesis: The Multitasking Approach of the First PEGylated Vascular Endothelial Growth Factor (CdtVEGF) from Brazilian Rattlesnake Venom
Source: Toxins (Basel). 2023 Jul 31;15(8):483. doi: 10.3390/toxins15080483 (PMC10467076; doi:10.3390/toxins15080483)
Supplement: Supplementary file 1 [file toxins-15-00483-s001.zip › toxins-2473181-supplementary.pdf]

toxins

MDPI

Supplementary Materials: Beyond Angiogenesis: The Multitasking Approach of the First PEGylated Vascular Endothelial Growth Factor (CdtVEGF) from Brazilian Rattlesnake Venom

Isabela Ferreira, Isadora Oliveira, Karla Bordon, Mouzarllem Reis, Gisele Wiezel, Caroline Sanchez, Luísa Santos, Norival Santos-Filho, Manuela Pucca, Lusânia Antunes, Daiana Lopes and Eliane Arantes

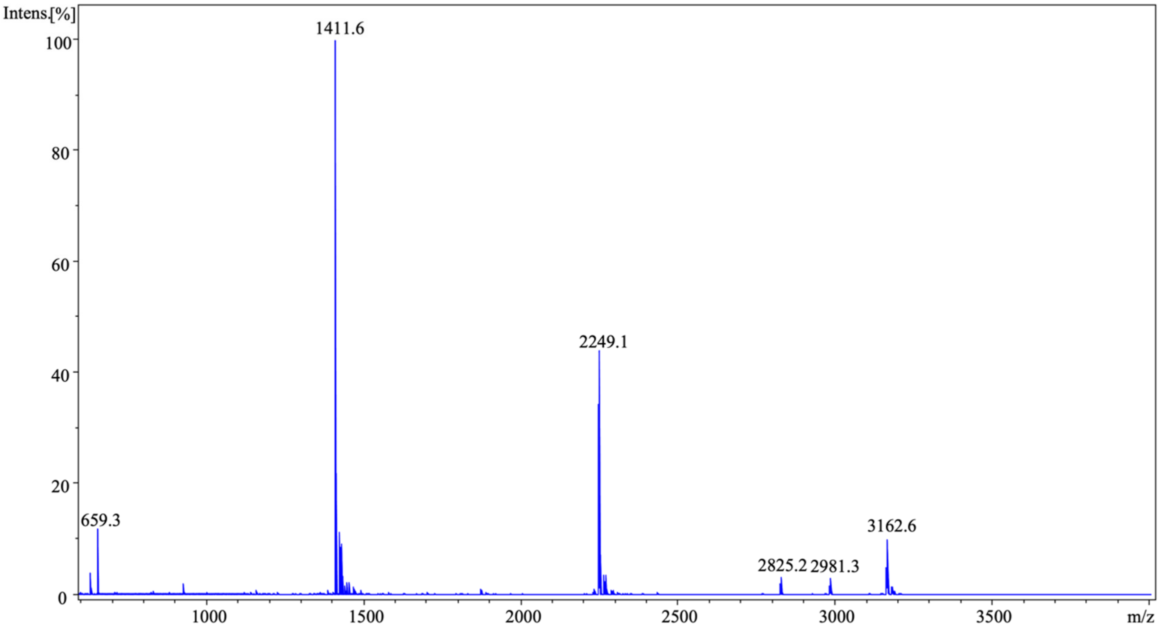

Figure S1. Peptide mass fingerprint of *Cdt*VEGF digested with trypsin.

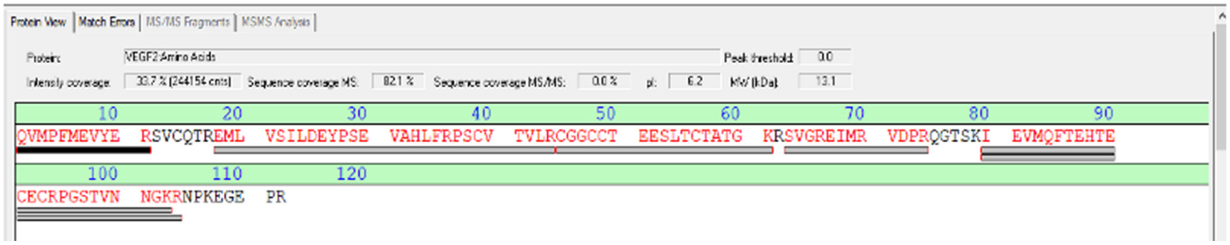

Figure S2. Sequence coverage of *Cdt*VEGF determined by peptide mass fingerprint. Black and gray bar: matching peptides.

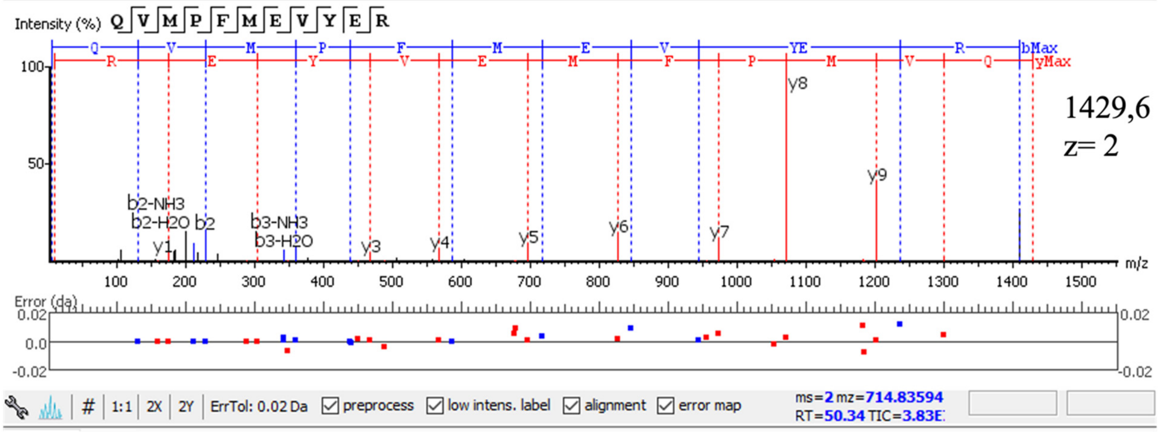
Intensity (%) QVMPFMEVYER
bMax
yMax
1429,6
z= 2
y8
y9
b2-NH3
b2-H2O b2
b3-NH3
b3-H2O
y1
y3
y4
y5
y6
y7
m/z
Error (da)
ErrTol: 0.02 Da
preprocess
low intens. label
alignment
error map
ms=2 mz=714.83594
RT=50.34 TIC=3.83E

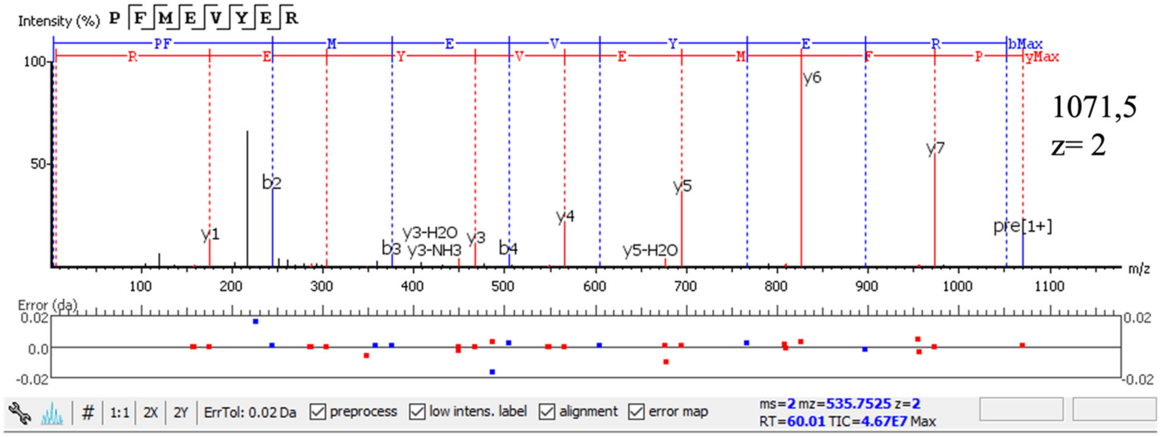
Intensity (%) PFMEVYER
bMax
yMax
1071,5
z= 2
y6
y7
b2
y5
y4
y1
y3-H2O
y3-NH3
b3
y3
b4
y5-H2O
pre[1+]
m/z
Error (da)
ErrTol: 0.02 Da
preprocess
low intens. label
alignment
error map
ms=2 mz=535.7525 z=2
RT=60.01 TIC=4.67E7 Max

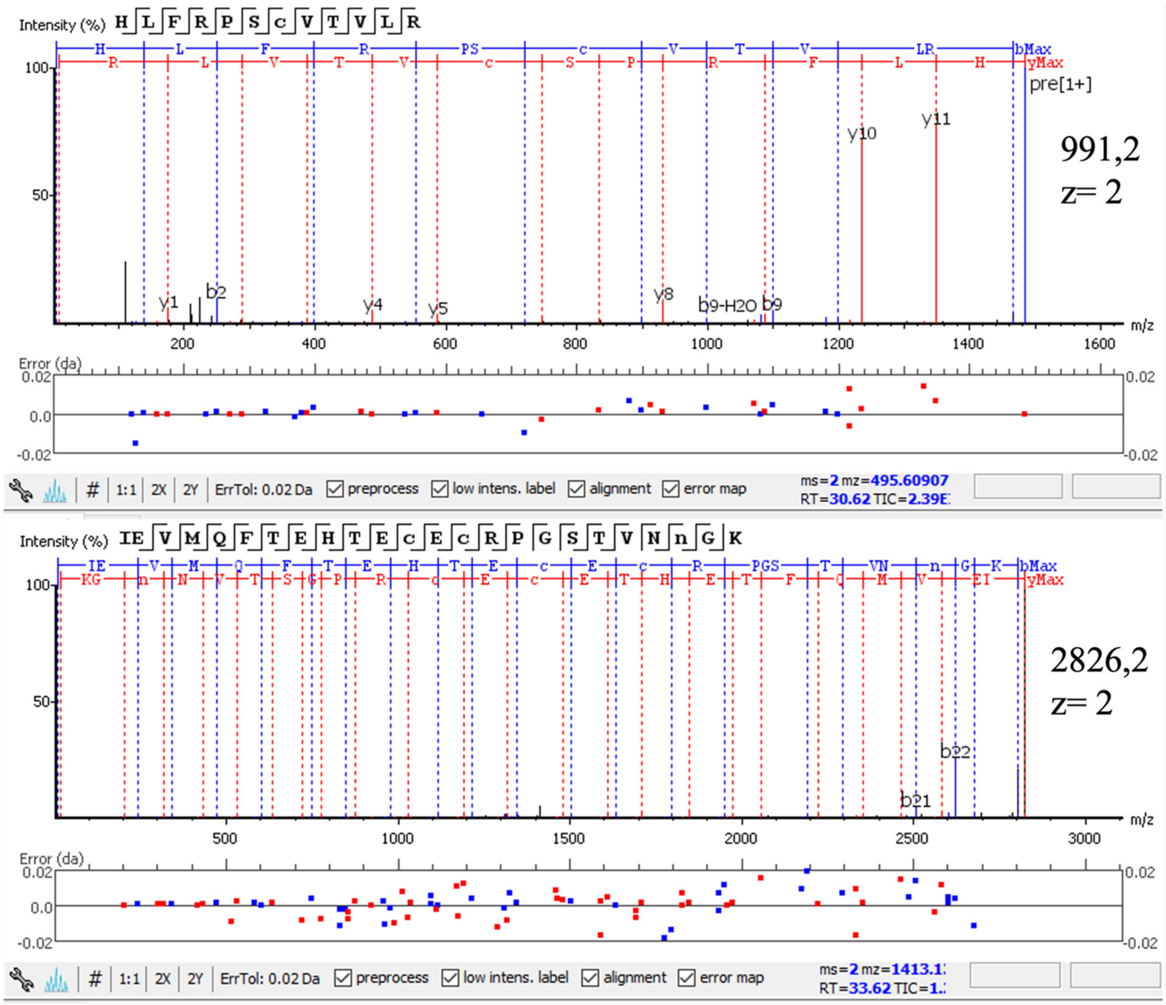
Intensity (%)
HLFRPScVTVLR
991,2
z= 2
pre[1+]
y10
y11
y1
b2
y4
y5
y8
b9-H2O
b9
m/z
Error (da)
ErrTol: 0.02 Da
preprocess
low intens. label
alignment
error map
ms=2 mz=495.60907
RT=30.62
IEVMQFTEHTEcEcRPGSTVNnGK
2826,2
z= 2
b22
b21
ms=2
RT=33.62

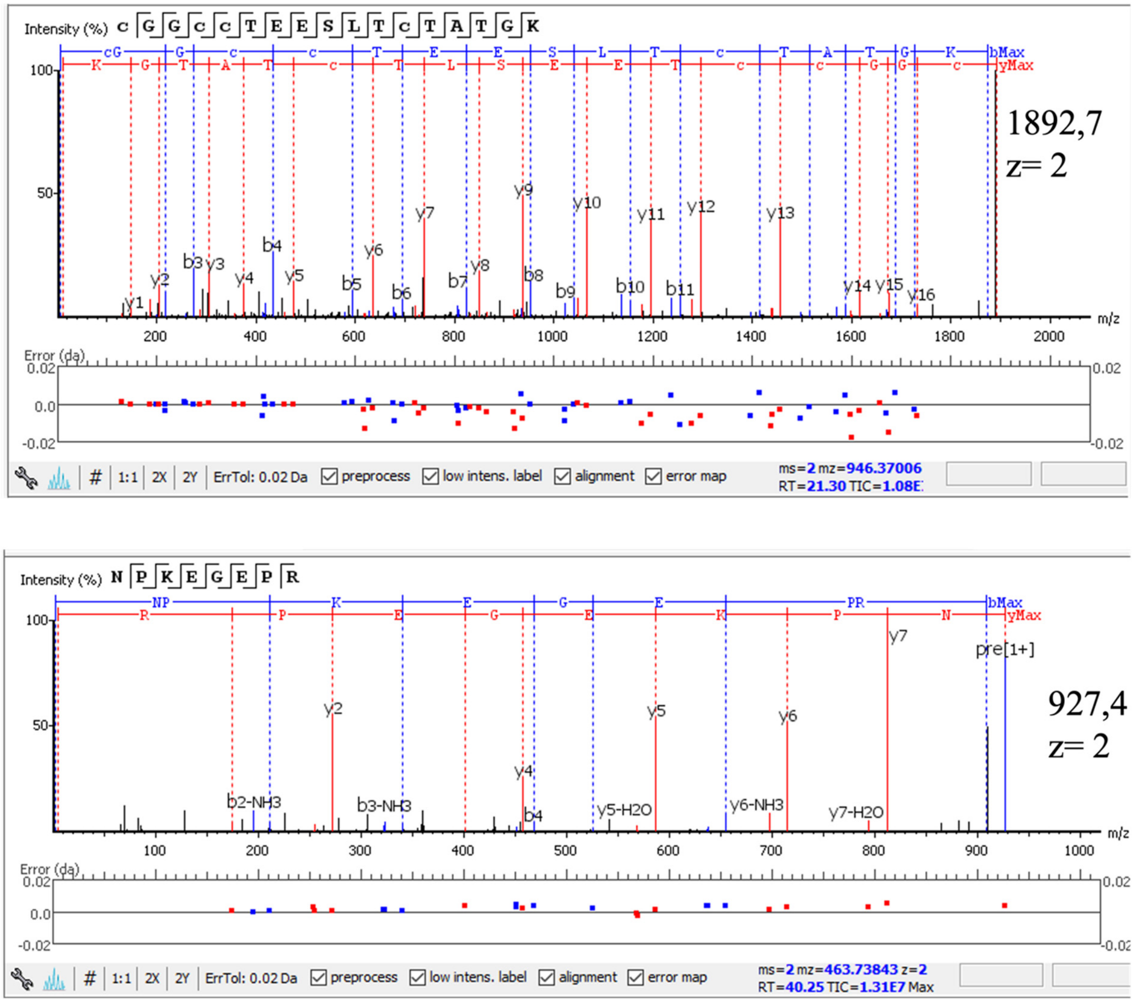

Figure S3. Mass spectra of matching peptides**.** The ion fragmentations were analyzed by PeaksStudioX software indicating y ions (blue) and b ions (red).

Table S1. Percentage of recovery from fractions obtained with PEGylation.

Sample	Purification step	Recovery (%)*
Fraction 2	Reversed-phase – C4	4.85
Fraction 3	Reversed-phase – C4	4.06
Fraction 4 (PEG-*Cdt*VEGF)	Reversed-phase – C4	76.32

* Recovery percentage calculated using Unicorn 5.2 software (GE Healthcare) according to the ratio between the area under the absorbance curve at 280 nm of each corresponding fraction and the sum of the areas of all fractions eluted.
